# Preparation and characterization of innovative cement mortar incorporating fatty acid/expanded graphite composite phase change material for thermal energy storage

**DOI:** 10.1038/s41598-024-67573-x

**Published:** 2024-07-17

**Authors:** Dongyi Zhou, Shuaizhe Xiao, Yicai Liu

**Affiliations:** 1 School of Energy and Mechanical Engineering, Hunan University of Humanities, Science and Technology, Loudi, 417000 China; 2 School of Mechanical and Energy Engineering, Shaoyang University, Shaoyang, 422000 China; 3 School of Energy Science and Engineering, Central South University, Changsha, 410083 China

**Keywords:** Energy science and technology, Materials science

## Abstract

To explore the application of phase change energy storage materials in building energy conservation, in this study, an innovative composite thermal energy storage cement mortar (CTESCM) was developed using lauric acid–palmitic acid/expanded graphite (LA-PA/EG) as the composite phase change material (CPCM). Seven different CTESCM test blocks with different CPCM mass contents were prepared. The thermal characterization of the CTESCMs was achieved using a differential scanning calorimeter (DSC), a thermogravimetric analysis (TGA), thermal conductivity tests, and heat storage/release tests. The physical behavior was assessed using density, mechanical performance was assessed using compressive strength, and the microstructure was observed using a scanning electron microscope (SEM). The results indicate that the phase transition temperature of the CTESCMs was lower than that of the LA-PA/EG CPCM, and the latent heat consistently decreased with the decrease of the CPCM mass content. With the addition of the CPCM, which had a low-density porous structure, the thermal conductivity, density, and compressive strength of the CTESCMs decreased. CTESCM with a mass fraction of 20%C (20% cement) CPCM can be used for building energy conservation such as floor radiation heating systems.

## Introduction

Energy shortage is a serious problem that the world must confronts in the 21-st century. In the field of energy conservation, building energy conservation accounts for a considerable share because of heating and cooling^1,2^. Therefore, it is necessary to pay attention to improving the thermal efficiency of buildings and the utilization of renewable energy^3,4^. Solar energy has enormous potential and is a type of renewable energy^5^. However, developing new building materials is also an effective means of improving the efficiency of building energy utilization and reducing building energy conservation.

At present, thermal energy storage technology is attracting increasing attention. Thermal energy storage technology comprises sensible heat thermal energy storage and latent thermal energy storage. Latent thermal energy storage technology is realized through the ability of phase change material (PCM) to store and release thermal energy to the environment, and it can control the internal temperature, thereby improving the energy efficiency of buildings^6–8^. Fatty acids are usually used in buildings as PCMs for latent thermal energy storage due to their excellent thermal performance^9,10^. However, their drawbacks, such as their low thermal conductivity and leakage during melting/freezing, hinder their application in buildings^11–13^. The common method is to combine PCMs with porous support materials to obtain form-stable CPCMs^14–17^. Proper support materials play a key role in the production of form-stable CPCMs. Therefore, various inorganic porous groups, polymer groups and microcapsules have been studied for the preparation of form-stable CPCMs^18–21^. Expanded graphite is widely used because of its porosity and high thermal conductivity. In form-stable CPCMs with expanded graphite as the supporting material, PCMs are adsorbed into the pores of the EG, which can not only prevent the leakage of liquid PCMs, but also increase the heat storage/release rate^22–24^.

The use of composite PCMs with building materials will effectively enhance the heat storage/release performance of building structures, ensuring better indoor thermal comfort^25^. Composite PCMs can be used with traditional building materials to form phase change building materials and absorb/release heat through changes in room temperature or by receiving solar radiation heat. Phase change walls, floors, roofs, mortar, etc., all belong to phase change energy storage building structures. Incorporating PCMs into coatings to form fixed CPCMs not only beautifies and protects the appearance of buildings but also achieves the goal of regulating indoor comfort through the heat storage and release performance of PCMs. Applying PCMs to glass windows can not only effectively improve indoor heat loss but also achieve applications in intelligent dimming and other fields through the optical effects generated by PCMs with temperature changes. Adding PCMs to cement or gypsum has a good effect on improving the heat storage/release performance of building structures. Therefore, the incorporation of PCM into building materials has been a topical subject in building energy conservation^26^. Many researchers have proposed different methods of using PCMs for building heat energy storage^27,28^. PCMs can be mixed with gypsum plaster board, concrete mortar, concrete, PVC panels, brick wall, blocks and bricks^29–31^. Zhang et al. prepared thermal energy storage cement mortar (TESCM) containing n-octadecane/EG CPCM. The research results showed that the density of TESCM was not significantly reduced, and that TESCM helps to reduce building energy conservation^32^. Li et al. developed a heat storage cement mortar (HSCM) incorporating expanded graphite (EG)/parafin CPCM. The research results showed that the heat storage coefficient of an HSCM plate is 1.74 times that of ordinary cement mortar and that it has good heat storage performance^33^. Zhang et al. prepared paraffin/MBFS C-PCMs containing blast furnace slag (BFS) as a support material and paraffin as a PCM, with a phase transition temperature of $50^{\circ }\hbox {C}$ and a latent heat of 36.4 J/g^34^. Niall et al. prepared two types of PCM-concrete composite panels containing microencapsulated paraffin and butyl stearate, and a proportion of the cement was replaced with ground granulated blast-furnace slag (GGBS) in the second type of plate. The research results showed that, although the addition of the PCM reduced the strength of the concrete, it significantly increased its storage capacity, and little benefit was gained by adding GGBS^35^. Hekimoğlu et al. developed new cement mortars containing different weight fraction of silica fume (SF)/capric acid–stearic acid eutectic mixture composite PCM (FSC–PCM). The research results showed that the mechanical properties of a certain mass fraction of new materials were within an acceptable range and had the ability to adjust indoor temperatures and reduce building energy conservation^36^. Selvasofia S. D. Anitha et al. synthesized an organic bio-nano-doped phase change material for building energy management. The results demonstrated that the inclusion of bio-nanoparticles above a 1.0% mass fraction significantly reduced the thermal storage capability of the PCM^37^. Research by Das, R et al. mainly focused on the use of phase change material (PCM) with low thermal conductivity and high heat storage in buildings, and it was observed that adding 10% micro-encapsulated phase change material (MPCM) to mortar decreased its thermal conductivity by 22%, thereby increasing the material’s thermal insulation^38^. Liu et al. prepared CF-LMT844/EP-MOC mortar. The optimal mixing amount of CF-LMT844/EP was found to be 20%, and the material maintainde good performance in practical application^39^. Yu et al. produced a composite phase change material to improve the freeze–thaw resistance performance of cement mortars, and the composite method and experimental results are helpful for understanding the freeze–thaw mechanism of cement mortars^40^. Cellat et al. developed a new microencapsulated bio-based PCM (mPCM), and 2 years of monitoring results showed energy savings of up to 13%^41^. Additionally, Cellat et al. investigated the suitability of butyl stearate as an intelligent concrete additive that is directly added to concrete structures, taking into account factors such as corrosion, rheology, and thermal performance^42^. Cellat et al. also prepared CA-LA and CA-MA PCMs, and the results showed that both PCMs were appropriate for use in self-compacting concrete mixtures used in buildings^43^.

Although some studies have been carried out on the thermal storage of building materials, cement mortars incorporating fatty acid binary eutectic mixtures/EG CPCM have not been developed in the existing literature, and evaluations of the heat storage effect have not been conducted. This study is a continuation of the previous work of the research team^44,45^. The objective of this study is to prepare a type of innovative thermal energy storage cement mortar with a good heat transfer ability and form-stability, compared with ordinary cement mortar, which can be used in floor radiation heating systems. In this paper, composite thermal energy storage cement mortars (CTESCM) with different LA-PA/EG CPCM mass content (0%, 5%, 15%, 20%, 30%, and 40% of cement) were prepared by mixing LA-PA/EG CPCM into cement mortar for the first time. Through a comparison with ordinary cement mortar, the thermal, physical and mechanical properties of the prepared CTESCMs were analyzed.

## Experiments

### Materials

Lauric acid (LA, 99% purity) and Palmitic acid (PA, 98.5% purity) were supplied by Shanghai Zhunyun Chemical Co., Ltd, China. The properties of the LA and PA are shown in Table 1. Expandable graphite (carbon content > 99%, expansion coefficient: 100 mL/g, 350 meshes) was purchased from Qingdao hengrunda graphite products Co, Ltd., China. The cement was ordinary Portland cement (PO 42.5), which was supplied by Shaoyang South Cement Co., Ltd, China. The chemical composition of the cement is shown in Table 2. The fineness modulus of the river sand was 2.50.

**Table 1:** Properties of LA and PA.

PCM	Temperature of phase change ($^{\circ }$C)	Latent heat of phase change (J/g)	Thermal conductivity (W/(mK))	Solid		Liquid	
				Density (kg/m$^3$)	Specific heat (kJ/(kg $^{\circ }$C))	Density (kg/m$^3$)	Specific heat (kJ/(kg $^{\circ }$C))
LA	42.4–44	174.9–186.4	0.147	1007	1.7	862	2.3
PA	58.9–64	185.4–212.1	0.162	989	1.9	850	2.8

**Table 2:** Chemical composition of cement.

Composition	SiO$_2$	Al$_{2}$O$_{3}$	Fe$_{2}$O$_{3}$	CaO	MgO	SO$_3$	Na$_2$O	K$_2$O	*Other*
Content (%)	8.46	33.38	3.12	42.35	2.29	9.53	0.21	0.05	0.61

### Preparation of the CPCM and heat storage mortar

**Figure 1:**
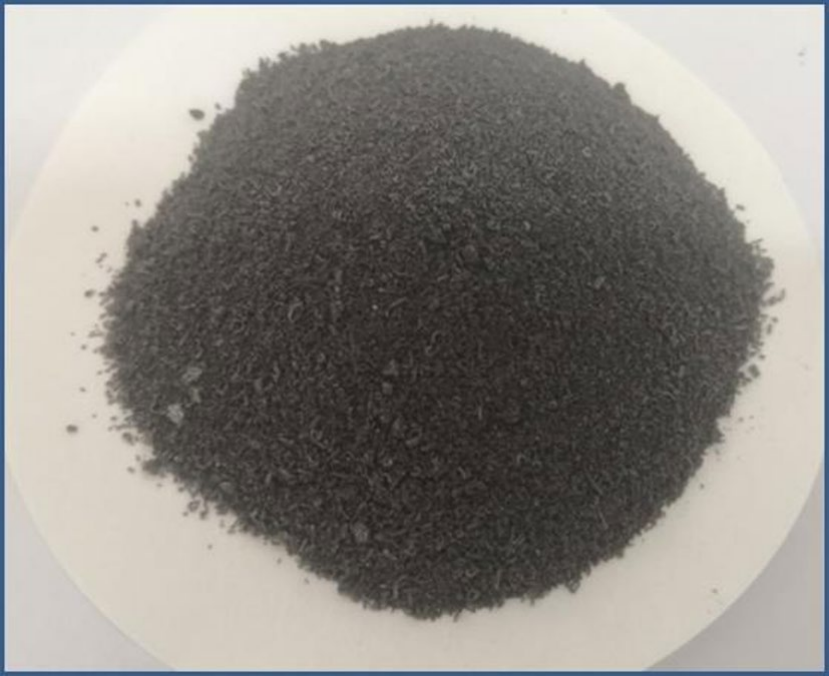
The LA-PA/EG CPCM.

The preparation of the LA-PA/EG CPCM in this work took into account previous works conducted by the authors regarding the preparation of fatty acid binary eutectic mixtures/expanded graphite for thermal energy storage^44,45^. The mass ratio of $m_{LA}$/$m_{PA}$ was 69.8/30.2 in the LA-PA PCM. The mass content of the LA-PA PCM in the LA-PA/EG CPCM was 92.4%. The prepared LA-PA/EG CPCM is shown in Fig. 1.

**Figure 2:**
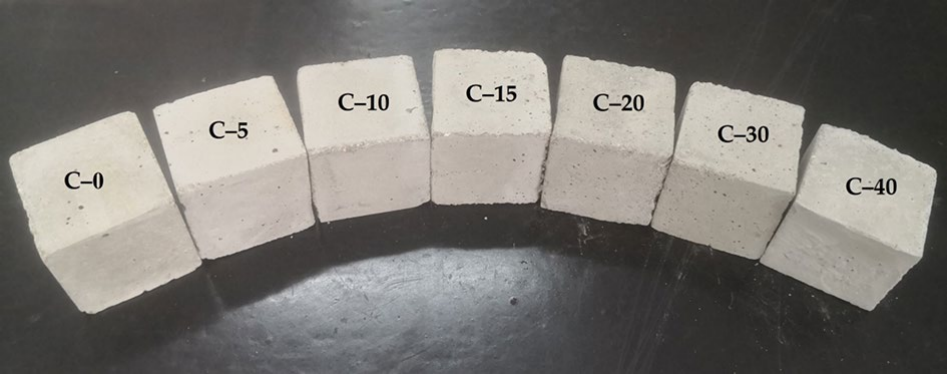
OCM and CTESCM test samples.

In this study, 7 different compositions were developed and evaluated. The LA-PA/EG CPCM content was fixed as a percentage of the total cement mass. Seven CPCM contents were used, namely, 0%C, 5%C, 10%C, 15%C, 20%C, 30%C and 40%C, and the corresponding test samples were labeled as C-0, C-5, C-15, C-20, C-30 and C-40, respectively. Table 3 shows the developed compositions. To prepare the CTESCMs, the LA-PA/EG CPCM was mixed with cement, sand, and water according to the mix proportions in Table 3. The mixtures were put into the container of the cement mortar mixer. The mixer was stopped after running for 2 min at a speed of 70 r/min, during which a small amount of water was added many times. Then, the viscous cement mortar was poured into the container, during which the bubbles were shaken out to prevent a large number of holes after molding. The prepared cement mortar was put into a mold with a size of 40 mm $\times$ 40 mm $\times$ 40 mm. After 48 h, the cement mortar was dried and peeled from the mold. After 28 days of curing, the blocks of CTESCMs (show in Fig. 2) to be tested were prepared after drying.

**Table 3:** Mix proportions of ordinary cement mortar (OCM) and the CTESCM.

Sample number	Cement (g)	Sand (g)	Water (g)	CPCM (g)	CPCM ($\%$)
C-0	200	400	100	0	0% cement
C-5	200	400	100	10	5% cement
C-10	200	400	100	20	10% cement
C-15	200	400	100	30	15% cement
C-20	200	400	100	40	20% cement
C-30	200	400	100	60	30% cement
C-40	200	400	100	80	40% cement

### Methods

The thermal properties of the LA-PA/EG CPCMs and CTESCMs were measured by using a differential scanning calorimeter (DSC, NETSZCH 214Polyma, Germany) at a heating rates of $5\;^{\circ }$C/min under a nitrogen atmosphere from 0 to $100\;^{\circ }$C. The surface morphological structure of the CTESCM samples was observed using a scanning electron microscope (SEM, phenom LE). The thermal stability of the LA-PA/EG CPCM was characterized via a thermogravimetric analysis (TGA, TA TGA5000IR, USA) at a heating rate of $10\;^{\circ }$C/min from 25 to $400\;^{\circ }$C. The long-term stability of the PCM, CPCM, and CTESCMs was determined by conducting a thermal cycling test. The DSC test was repeated after multiple thermal cycles to determine the thermal properties of the recycled PCM. A thermal conductivity tester (DRE-III, Xiangtan Xiangyi Instrument and Instrument Co., Ltd, China) was used to measure the thermal conductivity of the samples. For accelerated thermal cycling tests, high- and low-temperature test chambers (LINPIN, LRHS-101-LH, Shanghai Linpin Instrument Co., Ltd., China) were used with a heating rate of $2\;^{\circ }$C/min and a cooling rate of $2\;^{\circ }$C/min from 10 to $60\;^{\circ }$C. Compressive strengths was tested by using a microcomputer-controlled electronic universal test (WDW-100G, Jinan Chenda Testing Machine Manufacturing Co., Ltd, China) according to GB/T 50148-2014 (a standard for test methods for concrete blocks).

**Figure 3:**
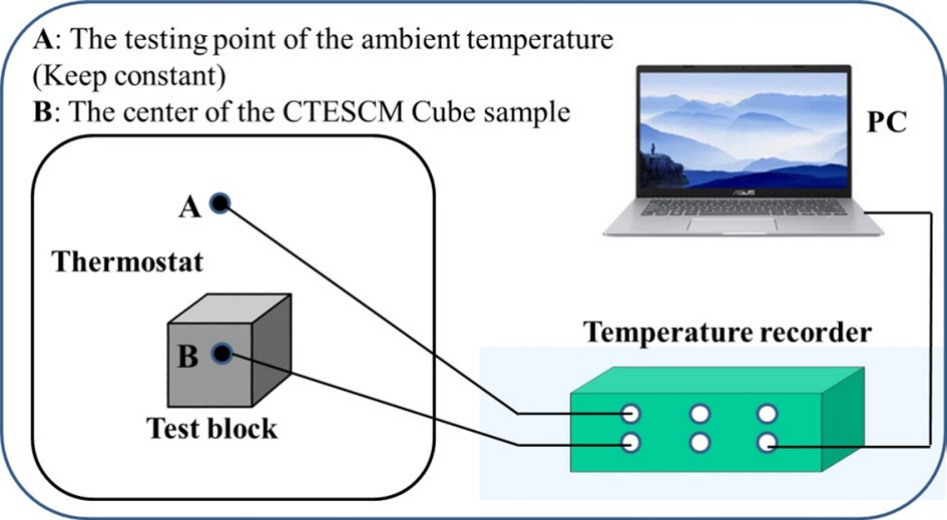
Setup of the heat storage and release tests.

Heat storage/release tests were carried out on the test blocks within a temperature range of 5–55 $^{\circ }$C. Figure 3 shows the setup of the heat storage and release tests. The size of the thermostat is 400 mm $\times$ 400 mm $\times$ 400 mm. A multi-channel temperature recorder logged the temperatures at points A and B. A thermocouple was placed in the center of the test block and filled with thermal conductive silica gel (a small hole for placing the thermocouple was reserved in the center of the test block). When performing the heat storage test, the test block was put into a thermostat of $5\;^{\circ }$C for cooling for 24 h so that the surface and center temperatures of the test block were both $5\;^{\circ }$C, and then it was put into a thermostat of $55\;^{\circ }$C for testing. The multi-channel intelligent temperature inspection instrument (XMT-JK800W, Yuyao Yitai Instrument Factory, China) was used to record the temperature changes of the test block, and the data were recorded every 10 s. The temperature of the heat release test ranged is from 55 to $5\;^{\circ }$C.

## Results and discussion

### The properties of the LA-PA/EG CPCM

**Figure 4:**
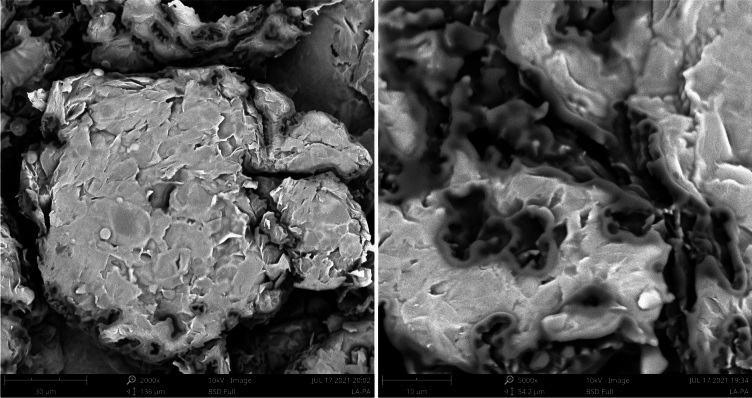
SEM images of the LA-PA/EG CPCM.

SEM images of the LA-PA/EG CPCM is shown in Fig. 4. The network pore structure of the EG was composed of graphite flakes and a large number of irregular pores. Under the capillary force of the pores, molten LA-PA could be easily adsorbed into the microporous structure of the EG. As can be seen in graph 4, LA-PA was uniformly adsorbed into the pore size of the EG, but the liquid LA-PA did not completely fill the microporous structure of the EG, and the prepared composite phase change material still retained the original worm-like morphology of EG. Therefore, the LA-PA phase change material was uniformly adsorbed in the honeycomb structure of the EG and had difficulty in leaking.

**Figure 5:**
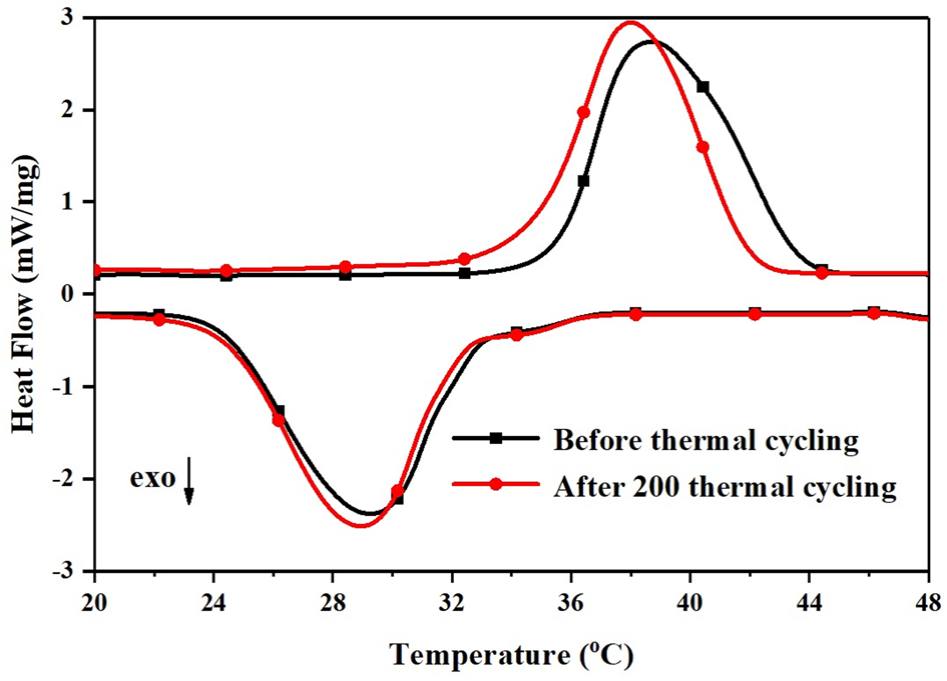
DSC curves of the LA-PA/EG CPCMs before and after 200 thermal cycles.

Figure 5 displays the LA-PA/EG CPCM’s DSC curves before and after 200 heat cycles, and the phase change temperature and latent heat are shown in Table 4. It can be seen that the phase change temperature of the LA-PA/EG CPCM is $35.5\;^{\circ }$C and that the phase change latent heat is 169.8 J/g. The supply and return water temperatures of floor radiant heating systems are generally between 35 and $45\;^{\circ }$C. Therefore, the LA-PA/EG CPCM has a suitable phase change temperature and a higher phase change latent heat for floor radiant heating systems. After 200 thermal cycles, the melting temperature and melting latent heat drop by $0.70\;^{\circ }$C and 1.4 J/g, respectively, and the modified values may be disregarded. This intimates that there is sufficient thermal stability in the LA-PA/EG CPCM.

**Table 4:** Thermal properties of the LA-PA/EG CPCMs before and after 200 thermal cycles.

PCM	Melting		Freezing	
	Temperature ($^{\circ }$C)	Latent heat (J/g)	Temperature ($^{\circ }$C)	Latent heat (J/g)
LA-PA/EG (before cycling)	35.5	169.8	33.0	155.2
LA-PA/EG (after 200 cycles)	34.8	168.4	32.5	153.1

**Figure 6:**
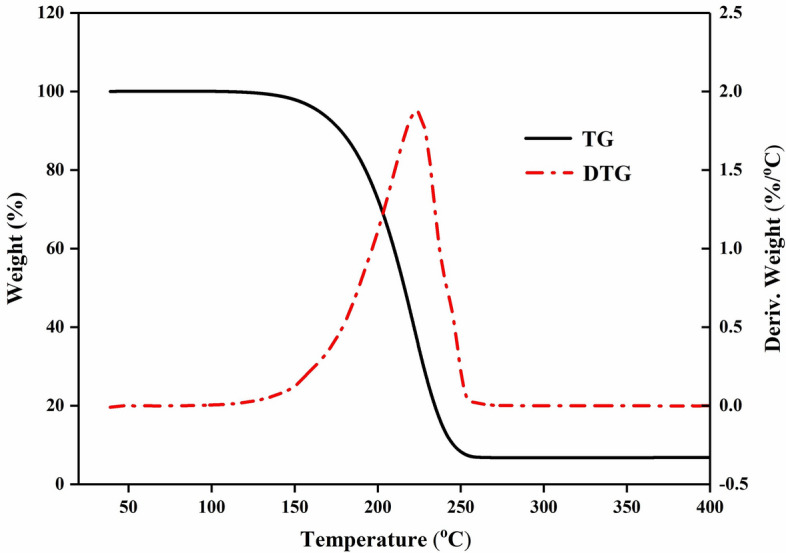
TGA curves of the LA-PA/EG CPCM.

Figure 6 displays the LA-PA/EG CPCM’s TGA curves. It can be seen that the LA-PA/EG CPCM starts to lose weight at around $138.8\;^{\circ }$C, reaches a maximum temperature of $225.9\;^{\circ }$C, and primarily loses weight in the range of 150–$230\;^{\circ }$C. At roughly $272.5\;^{\circ }$C, the LA-PA PCM fully volatilizes, leaving behind contaminants and non-volatile EG. Therefore, the LA-PA/EG CPCM has good thermal stability in application at $100\;^{\circ }$C.

### The thermal properties of the CTESCMs

#### DSC results

**Figure 7:**
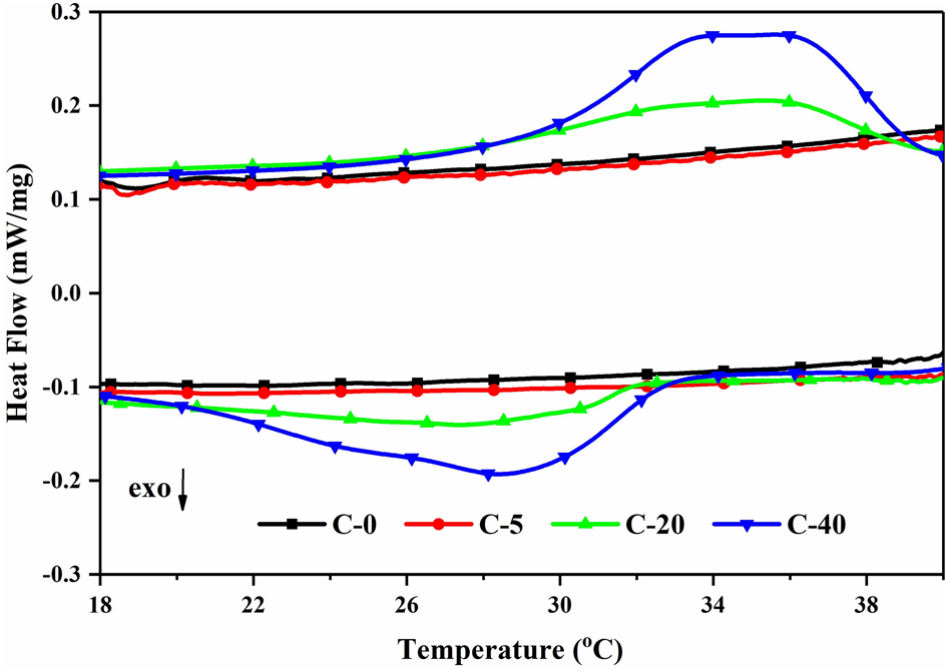
DSC curves of the CTESCMs with different CPCM mass contents.

Figure 7 shows the DSC curves of four types of CTESCMs with different LA-PA/EG CPCM mass contents. The corresponding DSC data of C-20 and C-40 are also given in Table 5. It can be seen that the phase change temperatures of the CTESCM are low compared with those of the LA-PA/EG CPCM, while the latent heat values of the CTESCMs consistently decrease according to the decrease in the CPCM mass content. The heating or cooling curve of C-0 (ordinary cement mortar) is almost a straight line, indicating that it is a normal inorganic material and has no heat storage capacity. C-5 has no obvious endothermic or exothermic peak due to its low CPCM content (5% cement), and its heat storage capacity is poor. When the mass of the LA-PA/EG CPCM in the CTESCM is relatively small, the peaks of the CTESCM DSC curves are not obvious. With the increase in the LA-PA/EG CPCM mass content, the DSC curves present obvious endothermic and exothermic peaks, such as the DSC curves of C-20 and C-40. In a comparison of the DSC curves, it can be clearly seen that sample C-40 is significantly different from sample C-5, and there are obvious absorption and exothermic peaks on the DSC curve of C-40, indicating that it has a certain heat storage capacity. The latent heat capacities of the CTESCM consistently decrease according to the decrease in the CPCM mass content.

**Table 5:** Thermal properties of the LA-PA/EG CPCM and C-20 and C-40 CTESCM.

Materials	Melting		Freezing	
	Temperature ($^{\circ }$C)	Latent heat (J/g)	Temperature ($^{\circ }$C)	Latent heat (J/g)
LA-PA/EG	35.5	169.8	33.0	155.2
C-20	26.3	7.77	32.0	7.17
C-40	28.8	14.48	32.9	12.60

#### Thermal conductivity

**Figure 8:**
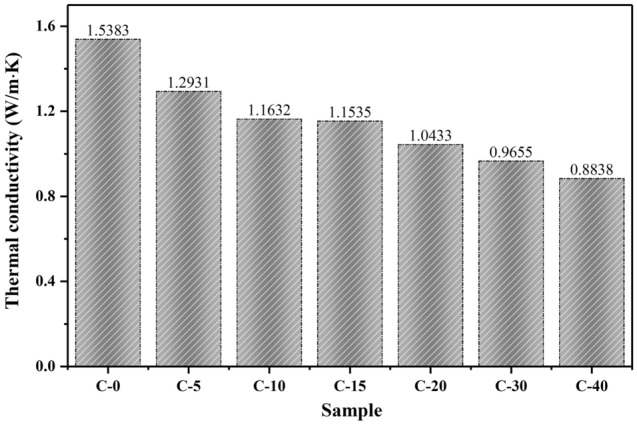
Thermal conductivity values of the CTESCMs with different CPCM mass contents.

Thermal conductivity is an important thermal property of the heat transfer ability of materials. The thermal conductivity test results of the prepared samples are presented in Fig. 8. The results show that the thermal conductivities of the CTESCMs decrease with the increase in the CPCM mass content. When the CPCM mass content increases from 0 to 5%C, 10%C, 15%C, 20%C, 30%C, and 40%C, the thermal conductivities decreases by 17.3%, 24.4%, 25.0%, 32.2%, 37.2%, and 42.5% respectively. A reason for this reduction may be the low thermal conductivity of the CPCM. After CPCM is mixed with cement mortar, the internal pores increase, and the binding between particles becomes loose, resulting in an increase in thermal resistance and a decrease in thermal conductivity. In this study, the prepared CTESCM is mainly used for floor radiation heating systems. Radiant heating floors are generally composed of floor slabs or floors adjacent to the soil, insulation layers, heating pipes, filling layers, ground layers, and waterproof layers (or moisture-proof layers). Due to the addition of CPCM, the thermal conductivity of the mortar decreases, resulting in a lower heat transfer coefficient for the floor. However, low thermal conductivity offers the possibility of providing a high thermal storage rate in building applications^28,46^.

#### Heat storage/release effect

**Figure 9:**
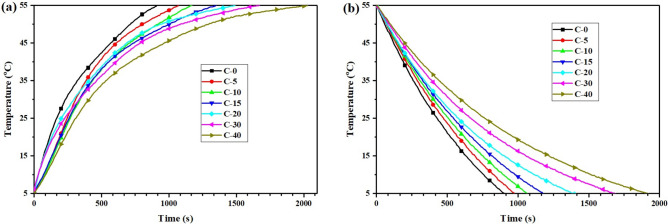
Heat storage (**a**) and release (**b**) curves of the CTESCMs with different CPCM mass content.

Figure 9 presents the heat storage and release curves of the CTESCM test blocks with different CPCM mass content. After adding the CPCM, the time taken for the temperature of the C-5 test block to rise from 5 to $55\;^{\circ }$C increased from 910 to 1490 s, which was 63.7% longer, and the time taken for the temperature to fall from 55 to $5\;^{\circ }$C increased from 900 to 1400 s, which was 55.6% longer. With the increase in the the CPCM mass content, the heat storage/release curves of the CTESCM test blocks became increasingly gentle. The time taken for the temperature of the C-40 test block to rise from 5 to $55\;^{\circ }$C extended from 910 seconds to 2030 seconds, which was 123.1% longer, and the time taken for the temperature to fall from 55 to $5\;^{\circ }$C extended from 900 to 1920 s, which was 113.3% longer. The reason for this phenomenon is that, when the temperature reaches the phase change temperature, phase transition will occur, thereby absorbing or releasing a large amount of latent heat, and this delays the internal heating or cooling rate of the sample. The addition of CPCM can significantly reduce the release rate of CTESCM and play a role in thermal energy storage.

#### Long-term stability

**Figure 10:**
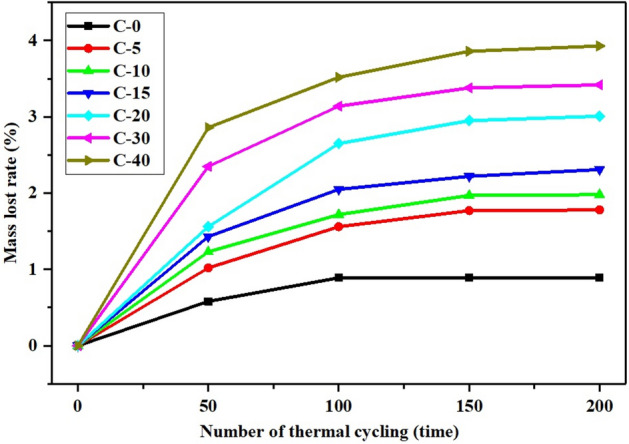
Mass loss rates of the CTESCMs with different CPCM mass contents after thermal cycling.

The long-term stability of the CTESCMs was determined by conducting accelerated thermal cycling tests. After every 50 cycles, the surface of the test block was cleaned with filter paper, and the mass of the test block with a balance was weighed. The number of thermal cycles was 200, and long-term stability was characterized by the mass loss of the CTESCM test block. Figure 10 presents the mass loss rates of the CTESCM test blocks with different CPCM mass contents after thermal cycling. The test results show that the mass loss of the CTESCM test blocks increased with the increase in the number of thermal cycles. For example, the mass loss rates of the C-20 test block were 1.56%, 2.65%, 2.95% and 3.01% when the number of thermal cycles was increased from 0 to 50, 100, 150, and 200. The mass loss rate of the CTESCM test block also increased with the increase in the CPCM mass content. After 200 thermal cycles, when the CPCM mass content of the CTESCM test block increased from 0 to 5%C, 10%C, 15%C, 20%C, 30%C and 40%C, the mass loss rate increased from 0.89 to 1.78%, 1.98%, 2.31%, 3.01%, 3.42% and 3.93%, respectively. The reason for this increase in mass loss is that the higher the content of CPCM, the more and larger the holes in the test block, and the more CPCM enters the test block. After many thermal cycles, the PCM undergoes multiple phase transformations. Although the PCMs were wrapped and shaped by the expanded graphite, it is inevitable that there will be a small amount of seepage. In addition, the mass loss may include a small amount of water evaporation loss. After 200 cycles, the maximum mass loss rate of the CTESCM test block was less than 4% of the total mass. Overall, the long-term stability of the CTESCM is good.

### Physical, mechanical, and microstructure properties of the CTESCMs

#### Density

**Figure 11:**
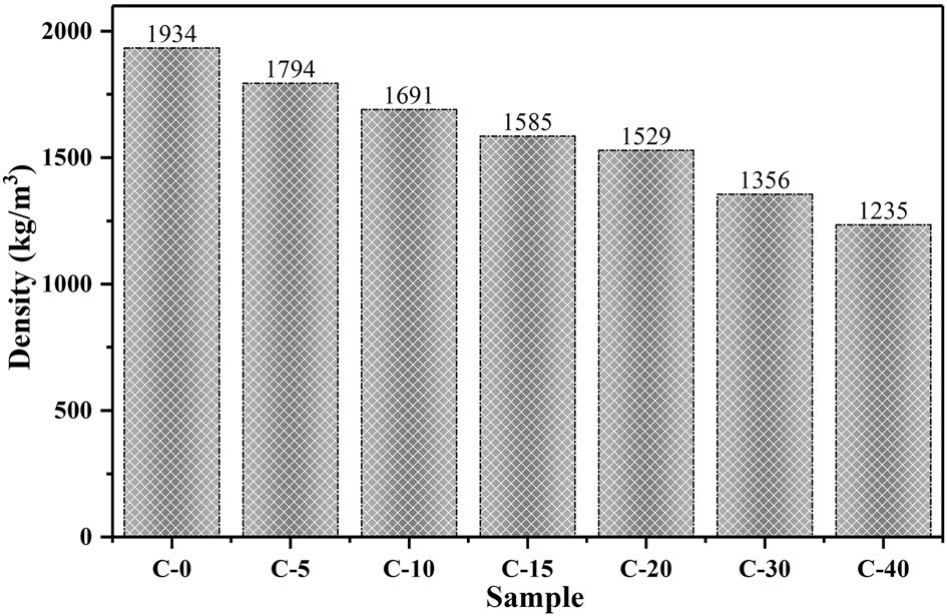
Density values of the CTESCMs with different CPCM mass contents.

Figure 11 presents the density values of the CTESCM test blocks with different CPCM mass contents. It can be observed that, with the increase in the CPCM mass content, the density of the CTESCM test blocks gradually decreased. There was an approximate linear relation between the density and the CPCM mass content. When the mass content of the CPCM increased from 0 to 5%C, 10%C, 15%C, 20%C, 30%C, and 40%C, the density decreased from 1934 kg/m$^3$ to 1794, 1690, 1585, 1529, 1356, and 1235 kg/m$^3$, decreases of 7.2%, 12.6%, 18.1%, 20.9%, 29.9%, and 36.1%, respectively. The density of the CTESCMs was significantly lower than that of ordinary cement mortar (C-0). There are two possible reasons for the reduction in density. One is related to the fact that the PCM occupied part of the microstructure of the CTESCMs, and the PCM had the low density. Conversely, the addition of CPCM will increase the presence of voids in aggregate or between aggregate particles, thus reducing the apparent density of the test block.

#### Compressive strength

**Figure 12:**
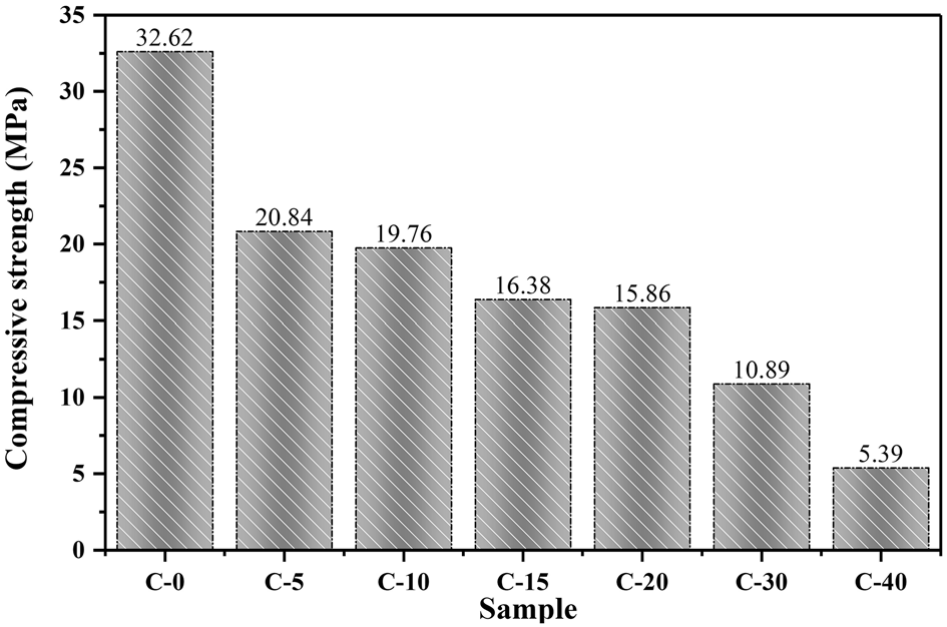
Compressive strength values of the CTESCMs with different CPCM mass contents.

The compressive strength results of the CTESCM test blocks with different CPCM mass contents are shown in Fig. 12. It was evident that, when the mass content of the CPCM increased, the compressive strength of the CTESCM test blocks decreased. The highest compressive strength (32.62 MPa) was reached in the C-0 test block without CPCM. When the CPCM mass content increased from 0 to 5%C, 10%C, 15%C, 20%C, 30%C, and 40%C, their compressive strength decreased from 32.62 MPa to 20.84, 19.76, 16.38, 15.86, 10.89, and 5.39 MPa, decreased of 36.1%, 39.4%, 49.8%, 51.4%, 66.6%, and 83.5%, respectively. When the CPCM mass content increased from 0 to 5%C, the compressive strength decreased sharply by 36.1% and then decreased slowly with the increase in the CPCM mass content. The reason for this is that the addition of CPCM will increase the existence of voids in or between aggregate particles, and the porosity of the CTESCM means that the strength of the aggregate in the cement mortar is weak. However, mortar’s compressive strength must not be lower than 15.0 MPa according the standard GB/T14902-2019. Among the samples, the test blocks with a CPCM mass content of less than 20%C meet the standard conditions in terms of compressive strength, such as the CTESCM C-20 sample (15.86 MPa).

#### Microstructure

**Figure 13:**
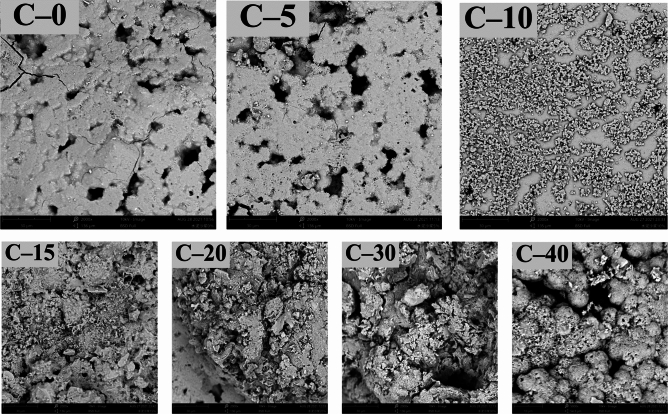
SEM images of the CTESCMs with different CPCM mass contents.

SEM images of the CTESCM test blocks with different CPCM mass contents are shown in Fig. 13. It can be observed that the internal crystals of the OCD C-0 test block without CPCM are mostly in the form of plate columns, with relatively tight bonding and low porosity. Compared with C-0, the crystals inside the other CTESCM test blocks are smaller, and the surface are covered with a layer of CPCM. The binding between the CPCM and mortar is loose, and the CTESCM has more internal pores. Furthermore, the higher the CPCM mass content in the CTESCM, the larger the porosity, which greatly weakens the binding force between mortar particles.

## Conclusion

In this study, an innovative cement mortar incorporating LA-PA/EG CPCM was prepared for building energy conservation such as floor radiant heating systems. The main conclusions drawn from the thermal, physical, mechanical, and microstructure properties of the CPCM, ORM, and CTESCMs are as follows:

1. The melting and freezing temperatures of the LA-PA/EG CPCM are $35.5^{\circ }$C and $33.0^{\circ }$C, while the latent heats are 169.8 J/g and 155.2 J/g, respectively. The LA-PA/EG CPCM has a suitable temperature and larger latent heat for floor radiant heating systems. The LA-PA PCMs are uniformly adsorbed in the honeycomb-like structure of the EG. The LA-PA/EG CPCM has adequate thermal stability.
2. The phase change temperature of the CTESCM is lower than that of the LA-PA/EG CPCM, and the latent heat consistently decreases according to the decrease in the CPCM mass content. The thermal conductivity of the CTESCM decreases with the increase in the CPCM mass content. The addition of CPCM can significantly reduce the storage/release rate of the CTESCM and play a role in thermal energy storage. After 200 cycles, the maximum mass loss rate of the CTESCM test block is less than 4% of the total mass, which indicates that the CTESCM has good long-term stability.
3. The density of the CTESCM test blocks gradually decreases with the increase in CPCM mass content. The compressive strength of the CTESCM is lower than that of the OCM, and the incorporation of CPCM can cause significant changes. Among the samples, the test blocks with a CPCM mass content of less than 20%C meet the the standard conditions in terms of compressive strength, such as the CTESCM C-20 sample (15.86 MPa). These behaviors are related to the addition of CPCM, which has a low density and a porous structure, in the CTESCM; this can be observed in the SEM images of the CTESCM.

The research presented above leads to the conclusion that CTESCM, a new energy-saving construction component for floor radiation heating systems, can be made by mixing LA-PACPCM with cement mortar. Using the CTSCM in building energy-saving systems can achieve significant advantages in energy conservation and carbon emissions, and it is a feasible and economical solution to improve building energy efficiency.

## Data Availability

The data that support the findings of this study are available on request from the corresponding author.
